# Non-destructive CT Method for Spatially Resolved Measurement of Elemental Content and Density of Li-B Alloys

**DOI:** 10.3389/fchem.2020.00781

**Published:** 2020-10-09

**Authors:** Haifeng Huang, Chen Wu, Zhijian Liu, Xiaopeng Zeng, Libao Chen

**Affiliations:** ^1^State Key Laboratory of Powder Metallurgy, Central South University, Changsha, China; ^2^Yichun Ganfeng Lithium Co., Ltd., Yichun, China

**Keywords:** CT scanning, spatially resolved measurement, non-destructive analysis, Li, Li-B alloy, density

## Abstract

Lithium-boron (Li-B) alloys play an important role in the fields of thermal batteries and Li metal batteries, where the electrochemical performance is highly dependent on microstructure homogeneity and the Li content. In this study, computed tomography (CT) scanning has been firstly used to study the elemental content and spatial distribution of Li in a Li-B alloy. For a commercial Li-B alloy, quantitative relationships between the CT values, [Hu], and the weight percent of Li, *w*_T−Li_, and the density, ρ_Li−B_, that is, [Hu] $=\frac{13563.8}{36.2\times w_{\text{T}-\text{Li}}-2.8}-1,016.2$ and [Hu] = 790.1 × ρ_Li−B_ − 1, 016.2, respectively. The experimental data were found to be in good agreement with current theory. The CT scanning method was non-destructive, and proved to be fast, highly accurate, and low-cost for the characterization of Li-B alloy ingots in terms of elemental composition, density, and uniformity.

## Introduction

Lithium-boron (Li-B) alloys play an important role in the fields of the thermal batteries and Li metal batteries, and this is attributed to several factors including high specific capacity, high specific power, the *in-situ* formed three-dimensional LiB skeleton, and similar electrochemical potential to Li (Guidotti and Masset, 2008; Duan et al., 2013; Cheng et al., 2014; Zhang et al., 2014; Liu et al., 2018; Zhong et al., 2018; Wu et al., 2020). The electrochemical performance of Li-B alloys is highly dependent on the homogeneity of the microstructure, the Li content, and the presence of defects (such as holes, cracks, impurities). For example, the existence of cracks may lead to a direct failure of the thermal battery, which often has to operate under harsh environmental conditions (high acceleration, spin, shock, and vibration) and thus reliability is a strict requirement (Guidotti, 1995). Moreover, the Li content will affect the discharge capacity, thus highly accurate measurement is required. However, the prepared Li-B alloys are usually unstable in nature due to the following factors: (1) The exothermic nature of the preparation process for Li-B alloy usually results in local overheating, which worsens the uniformity of the prepared samples. (2) During the final solidification process, shrinkage pores are formed within the alloy ingot. The pores, which originate from the inconsistent cooling rate from the outside to the inside of the ingot, will affect the local distribution of compounds. (3) Boron powder, for example, tends to sink due to gravity despite the use of vigorous stirring, which, in turn, results in volumetric shrinkage of the ingot and the synchronized filling of liquid Li into the skeleton structure. Therefore, knowledge of the spatial distribution of the alloying elements, the material density, and the internal defects is a critical factor for determining whether the quality of the alloy meets the necessary standards for the intended application.

Bulk analysis techniques such as inductively coupled plasma atomic emission spectrometry (ICP-AES) are considered unsuited to the analysis of Li-B alloys due to its destructive nature and the fact that there is a 2–4% deviation from the actual content of Li (Niu et al., 2014a,b; Ren et al., 2019). Morphological analysis techniques such as scanning electron microscopy (SEM) are also inappropriate for assessing the spatial distribution of the defects or LiB fibers because it is not possible to cut the ingot into numerous parts for observation. Energy dispersive X-ray spectrometry is not applicable to the determination of the spatial distribution of Li due to the low detector resolution for measurement of the X-ray energy emitted by excited-state Li atoms. In addition, all the above techniques are destructive in nature, hence the Li-B alloy samples cannot be further used after analysis. Moreover, the high reactivity of Li-B alloy with air and water complicates the characterization process. Therefore, new methods to effectively and non-destructively evaluate the quality of Li-B alloys are urgently needed.

Recently, computed tomography (CT), as a non-destructive analysis technique, has become an effective and common method for characterization of the internal structure of materials used in battery technology. The CT technique is based on the Beer-Lambert law, whereby the attenuated X-rays are related to the thickness and absorption coefficient of the material. The collected signals for thin slices of the sample are processed and transformed into images, which reflect the content and density of each component in the material, in such a way that the CT technique can display a distribution map of the components. Generally, there are three types of CT scanner in use: the industrial CT, the micro-CT, and the medical CT. For this study, the medical CT scanner has been selected based on the following considerations: (1) The industrial CT, with an X-ray energy up to the MeV level, can ensure a certain penetration depth for common steel materials (Saewert et al., 2003; Yin et al., 2011). However, the linear absorption coefficient (LAC) of Li-B alloy (0.05 cm^−1^) is far lower than that of iron, 0.47 cm^−1^ (under the condition of the monochromatic X-ray energy of 1 MeV photons) (Hubbell and Seltzer, 1995), and the Li-B alloy exceeds the detectable resolution area of the industrial CT. (2) The micro-CT with a micron-level resolution is usually suitable for the detection of small samples with dimensions of a few centimeters (Ho and Hutmacher, 2006); while the Li-B alloy ingot may reach more than 20 cm. (3) Fortunately, the average LAC of the Li-B alloy (0.12–0.16 cm^−1^) is close to that of water (0.19 cm^−1^, under the condition of the monochromatic X-ray energy of 73 keV photons). Also, the similar density (0.85–0.95 g cm^−3^) to human tissue and wide availability and maturity of medical CT systems facilitates use of this instrument for analysis of the Li-B alloy (Nymark and Nasstrom, 1992). To the best of our knowledge, there are no such reports on this application, hence application of the CT technique to the analysis of Li-B alloy for the first time is meaningful.

An overview of the principles of the CT scanning method as applied to Li-B alloys is first given, including the fundamental equation linking the CT value and the Li-B alloy density as well as the Li content. The basic method for identification of phase composition, and selection and calculation of the relevant experimental parameters are discussed in detail. Finally, verification of theory and an actual measurement are given. The results concerning the spatial distributions of defects, the Li content, and the density of a Li-B alloy are presented and evaluated. The establishment of this new method will ensure the stability and the reliability of Li-B alloys and promote the use of Li-B alloys in the Li metal battery and thermal battery fields.

## Materials and Methods

The preparation of the Li-B alloy is discussed in detail in a previous work (Ren et al., 2019). The elemental weight ratio for the Li ingot (purity 99.9%), boron powder (purity 99%), carbon powder (purity 99.9%), and Mg powder (purity 99.5%) was 61/27.5/7.5/4 as reported in a patent (Liu, 2007). The sizes of the prepared Li-B alloy ingots were about ϕ 140 × 180 mm^3^ and ϕ 170 × 240 mm^3^. To eliminate the holes generated during the synthesis of the Li-B alloy (Liu et al., 2001), the ingot was cold isostatic pressed (CIP) at a pressure of 200 MPa for 5 min.

An X-ray diffractometer (XRD, Rigaku D/max 2550PC) equipped with a Cu-*K*α radiation source (λ = 1.54056 Å), was used to measure the phases of the Li–B alloy. A scan speed of 8° min^−1^ and an interval of 0.02° were adopted. A scanning electron microscope (SEM, JSM-6360LV) was used to observe the microstructure of the Li-B alloy. To observe the skeleton morphology of the Li-B alloy, the sample was soaked in a solution of tetrahydrofuran (THF) with 8 wt.% naphthalene (C_10_H_8_) for 1 week to remove the Li matrix (Kilroy and Angres, 1979).

**Figure 1:**
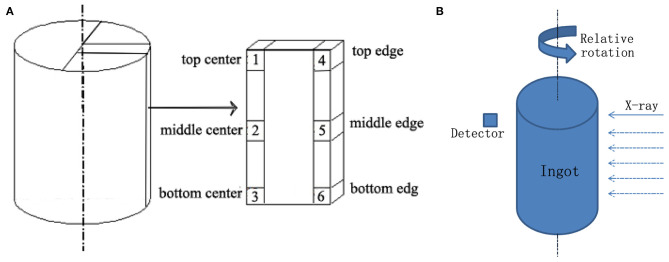
**(A)** Samplings for Archimedes density test and **(B)** Schematic of the CT scanning process.

The density of the Li-B alloy, ρ_Li−B_, was measured by the Archimedes method. The test was conducted in liquid paraffin and the density was calculated according to the equation: $\rho_{\text{Li}-\text{B}}=\frac{w_{1}}{w_{1}-w_{2}}\times \rho_{\text{LP}}$, where ρ_LP_ is the density of the liquid paraffin, and *w*_1_ and *w*_2_ are the weights of the Li-B alloy block in the glove box filled with dry air and in the liquid paraffin, respectively. For the measurement, the blocks of 1 cm^3^ were cut from different positions of the ingot, as shown in Figure 1A.

The ICP-AES (ICAP 7000) with a wavelength coverage of 166 nm to 847 nm and a spectral bandwidth of 7 pm at 200 nm was used to determine the composition of the Li-B alloy. To prepare the sample for ICP-AES analysis, a Li-B alloy block of 1 cm^3^ was first reacted with water and then hydrochloric acid was added until there was no precipitate in the solution. Subsequently, the solution was continuously diluted until the concentration reached 0.01 mg ml^−1^ (1 mg Li-B in 100 ml high purity water).

A conventional CT scanner (PHILIPS Brilliance^TM^ (16) featuring a scan voltage of 120 kV and a current of 200 mA, was employed for the CT analysis. In the CT test, the Li-B alloy ingot was protected with aluminum plastic foil to prevent oxidation and placed into a spiral CT scanning bed. A schematic of the CT test method is presented in Figure 1B. The slice thickness was 1 mm, and the spacing between slices was 0.5 mm.

## Results and Discussion

### Phase Composition and Microstructure

**Figure 2:**
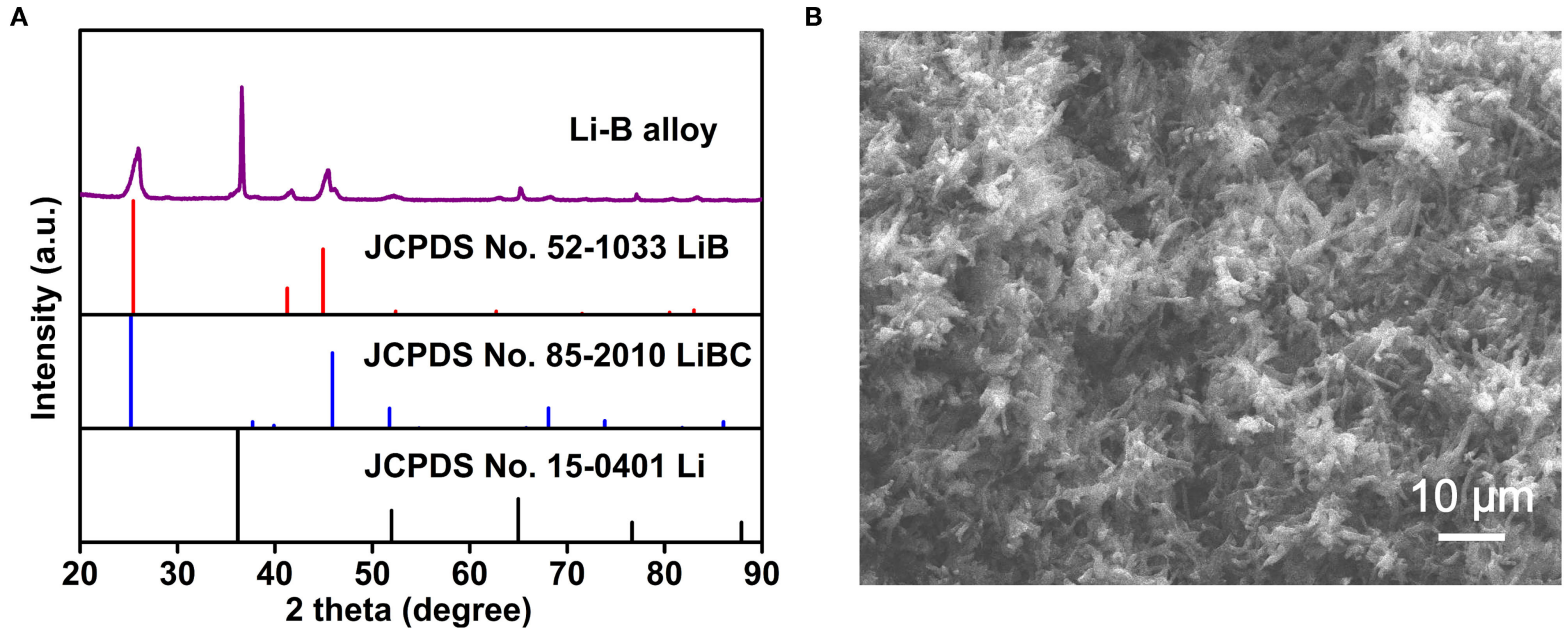
**(A)** The XRD pattern for the Li-B alloy. **(B)** The SEM image for the Li-B alloy after Li dissolution.

The XRD pattern of the Li-B alloy is illustrated in Figure 2A. The characteristic peaks corresponding to Li (JCPDS card No.15-0401), LiB (JCPDS card No.52-1033), and LiBC (JCPDS card No.85-2010) can be clearly observed. It is noteworthy that all the diffraction peaks belonging to the Li phase shifted to lower angles than those for the standard Li phase (JCPDS card No.15-0401), indicating the lattice expansion induced by the dissolved Mg atoms in the body-centered cubic (bcc)-Li, and resulting in a Li-Mg solid solution (SS). The Li-B alloy, therefore, consisted of a Li-Mg SS and a compound of LiB, as well as a minor compound of LiBC.

As shown in Figure 2B, the SEM image of the Li-B alloy after Li matrix dissolution reveals the skeleton-like microstructures (SLM) consisting of LiB and LiBC compounds with a size of about 10 μm. The SEM also indicates that Li is contained within the skeletal pores.

### The Stability and Accuracy of the CT Method

The CT scanning process essentially reflects the attenuation of X-rays by each component in a scanned sample. The CT values of water and air are defined by Hounsfield as 0 and−1,000 Hu, respectively (Nymark and Nasstrom, 1992). So the CT value, [Hu], for an element X is given by

(1) $$\begin{array}{r} \left[\text{Hu}\right]=\frac{\mu_{\text{x}}-\mu_{{\text{H}}_{2}\text{O}}}{\mu_{{\text{H}}_{2}\text{O}}}\times 1,000 \end{array}$$

where μ_*H*_2_*O*_ and μ_*x*_ are the LAC of water and the element X, respectively. According to the definition of the CT value, the LAC of water is about 0.19 cm^−1^ when adopting monochromatic X-ray photons of 73 keV (Nymark and Nasstrom, 1992; Hubbell and Seltzer, 1995). Therefore, the samples in this study are all measured for the condition of incident X-ray photons of 73 keV.

**Figure 3:**
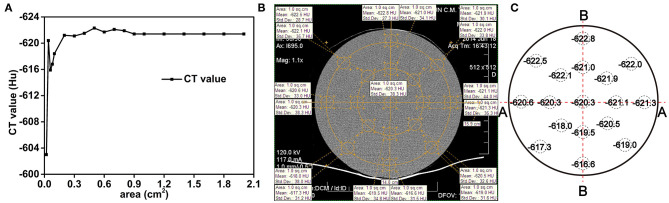
**(A)** The effect of the selection size area on the fluctuation of the CT values for a pure and uniform Li ingot. **(B)** Sampling method for CT scanning, **(C)** The CT values.

The spatial resolution of the CT system was set as 0.5 × 0.5 × 1.0 mm^3^, which corresponds to a measurement voxel. Given that the spacing between slices was set as 0.5 mm, the actual volume represented by each voxel was 0.125 mm^3^. In medical or industrial applications, the CT technique divides the sample into regions of a particular size and averages the values of all the voxels within that region as the representative value of the selected region which is called the “region of interest (ROI).” What is imperative is the relative differences of representative values in adjacent ROIs, but the size of the ROI needs to be determined and verified at first. This first step is called the accuracy and stability test of the CT method. If the ROI is too small, the x-ray counting differences from the statistical fluctuations among adjacent ROIs will be too large to identify different components. However, sharp fluctuations in the CT values will result in a high background and no effective information will be obtained. With too large ROIs, the average value may not capture important fine-scale details of the components. Therefore, a dense and uniform metal Li ingot of the same shape and size as the Li-B alloy ingot under study is first used as a standard sample to characterize the CT system accuracy and determine the key experimental parameters. Figure 3A shows the fluctuation characteristics of the average CT value for different sizes of the selection area. It is clear that the smaller the area, the larger the fluctuation of the CT value. The CT value tended to be stable after the size reached approximately 1 cm^2^, which is the value, therefore, chosen as the minimum selection area for the CT digital measurement.

The results for the distribution of the CT values in different regions of the pure Li ingot are presented in Figures 3B,C. It can be seen from Figure 3C that the CT values were relatively stable and especially so in the cross section (A-A), the variability in the CT values being within 1 Hu from the edge to the center. The fluctuations of the CT values in the vertical direction (B-B), which were influenced by the CT scanning bed, were slightly larger than that in the cross section (A-A), and were within 5 Hu from the edge to the center. This result indicated that CT scanning may be used as an accurate and reliable method for the determination of the homogeneity of the Li-B alloy. To obtain higher accuracy, the CT data used for the calculation was only taken from the cross section. Because the density distribution of the alloy ingot was axisymmetric, the accuracy of the test data was subject to fluctuation in the cross section CT value.

### Measurement of Defects via the CT Method

**Figure 4:**
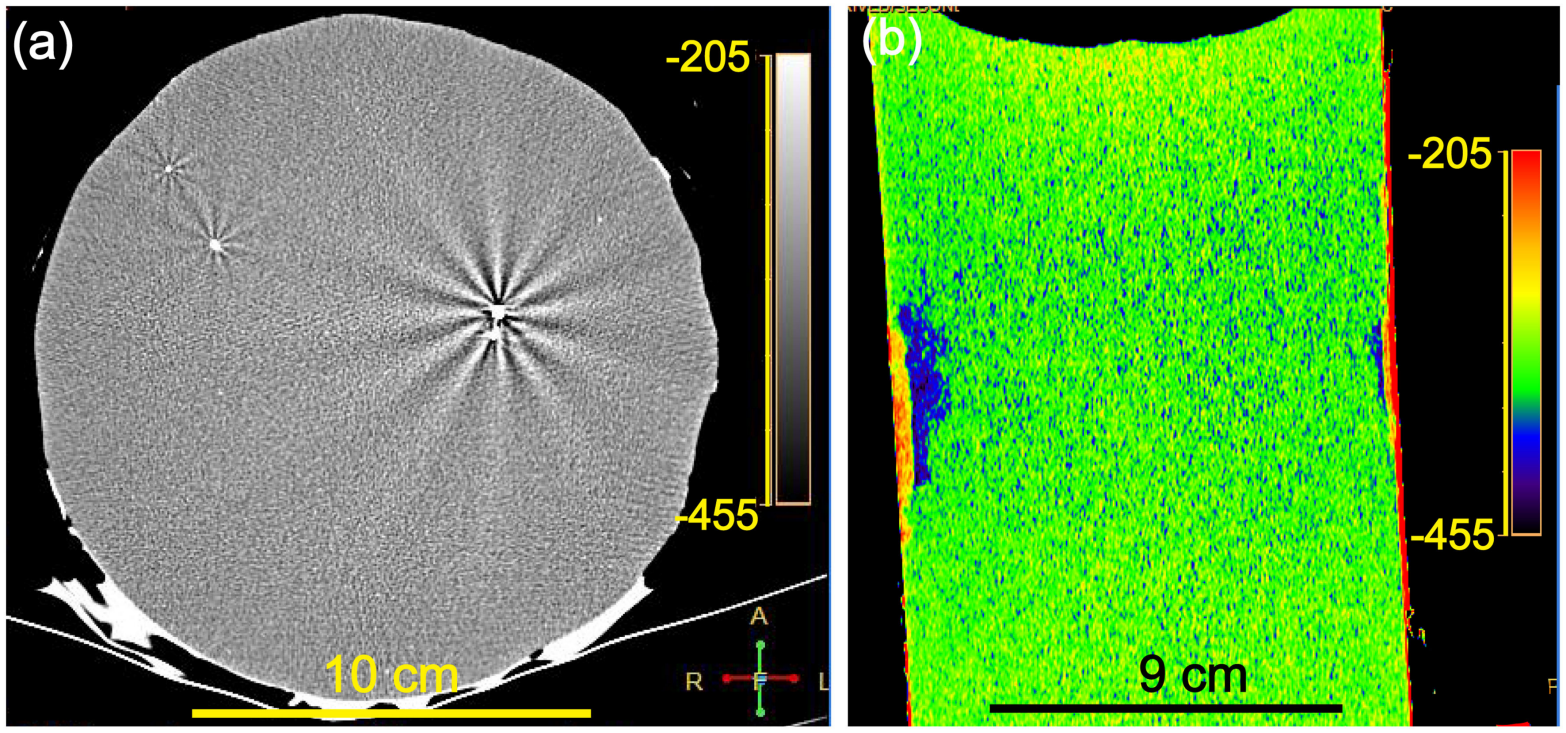
**(a)** CT image for a high-density heterogeneous crystal impurity in Li-B alloy. **(b)** CT image for the Li-B alloy at the z-axial section.

The CT image of a high-density heterogeneous crystalline impurity (2,950 Hu) in the Li-B alloy sample, which is in sharp contrast with that of the Li-B alloy, is presented in Figure 4a. This impurity may originate from the raw powder or from the sample preparation process for the ingot (e.g., the stirring process). The interaction between this impurity and the X-rays produce a second order diffraction phenomenon, which would not in theory occur in the alloy itself due to presence of only light elements in the alloy (i.e., Li and B). The radioactive secondary X-rays which would be emitted from the impurity were again absorbed by the surrounding Li and B atoms, which increased the LAC, so the CT value became larger and appeared as bright radial stripes on the CT image. The presence of such defects in Li-B alloys would not be detectable without the availability of CT analysis technology and clearly their presence in the alloy represents a serious potential problem affecting mechanical processing and electrochemical performance, which would not be acceptable for a finished Li-B alloy product. As shown in Figure 4b, the CT image of the axial section reveals that there is a big difference in the actual X-ray absorption capacity of the material in the different regions as evidenced by the different colors. The different colors represent the wide variation in CT values, reflecting the changes of density. The closer the color is to red, the larger the CT value and the higher the density, reflecting the presence of more framework phase components (LiB and LiBC). The closer the color is to purple, the smaller the CT value and the lower the density, reflecting the presence of more matrix components (Li-Mg SS). Thus, CT scanning provides a powerful means to measure and image the uneven spatial distribution of the components, which may reflect uneven shrinkage during ingot preparation and differences in the elemental composition and the porosity. Further commentary on these differences will be elaborated later.

### Spatially Resolved Measurement of Li Content and Density via the CT Method

This section is focused on the quantitative relationships between the CT value and the Li content as well as the density of the Li-B alloy. Then, verification of the relationships is confirmed to demonstrate the feasibility of using a medical CT scanner for spatially resolved measurement and imaging analysis of Li-B alloys. Also, the comparison of the CT method with other methods is undertaken to illustrate the superior performance of the CT method.

#### The Relationship Between CT Value and the Li Content or Density

For the Li-B alloy, Equation (1) can be re-written as

(2) $$\begin{array}{r} \left[\text{Hu}\right]=\frac{\mu_{\text{Li}-\text{B}}-\mu_{{\text{H}}_{2}\text{O}}}{\mu_{{\text{H}}_{2}\text{O}}}\times 1,000 \end{array}$$

where μ_Li−B_ is the LAC of the Li-B alloy. It is well-known that the LAC is equal to the product of the density, ρ, and the mass absorption coefficient (MAC), μ_m_. The μ_Li−B_ is, then, given as follows

(3) $$\begin{array}{r} \mu_{\text{Li}-\text{B}}=\rho_{\text{Li}-\text{B}}\cdot \mu_{\text{m}-\left(\text{Li}-\text{B}\right)} \end{array}$$

where ρ_Li−B_ is the density of the Li-B alloy. First, given that only LiB and Li phases are involved, the density of the Li-B alloy can be written as follows:

(4) $$\begin{array}{r} \rho_{\text{Li}-\text{B}}=1/\left(\frac{{\text{w}}_{\text{Li}}}{\rho_{\text{Li}}}+\frac{{\text{w}}_{\text{LiB}}}{\rho_{\text{LiB}}}\right). \end{array}$$

where *w*_Li_ and *w*_LiB_ are the mass fractions of Li metal and LiB, respectively, and ρ_Li_ and ρ_LiB_ are the densities of Li metal and LiB, respectively. Also, *w*_LiB_ = 1–*w*_Li_ for the situation when only LiB and Li phases are present in the alloy. In addition, the MAC of the Li-B alloy, μ_m−(Li−B)_, is given by

(5) $$\begin{array}{r} \mu_{\text{m}-\left(\text{Li}-\text{B}\right)}={\text{w}}_{\text{Li}}\cdot \mu_{\text{m}-\text{Li}}+{\text{w}}_{\text{LiB}}\cdot \mu_{\text{m}-\text{LiB}} \end{array}$$

where μ_m−Li_ and μ_m−LiB_ are the MACs of Li metal and LiB, respectively. Combining Equations. (2) – (5), Equation (6) is derived as follows:

(6) $$\begin{array}{l} \left[\text{Hu}\right]=\frac{\left({\text{w}}_{\text{Li}}\cdot \mu_{\text{m}-\text{Li}}+{\text{w}}_{\text{LiB}}\cdot \mu_{\text{m}-\text{LiB}}\right)/\left(\frac{{\text{w}}_{\text{Li}}}{\rho_{\text{Li}}}+\frac{{\text{w}}_{\text{LiB}}}{\rho_{\text{LiB}}}\right)-\mu_{{\text{H}}_{2}\text{O}}}{\mu_{{\text{H}}_{2}\text{O}}}\\ \;\times 1,000 \end{array}$$

Substituting *w*_LiB_ = 1–*w*_Li_ in Equation (6), the CT value is

(7) $$\begin{array}{l} {}[\text{Hu}]\text{=}\frac{[{\text{w}}_{\text{Li}}\cdot \mu_{\text{m}-\text{Li}}\text{+}(\text{1}-{\text{w}}_{\text{Li}})\cdot \mu_{\text{m}-\text{LiB}}]\text{/}(\frac{{\text{w}}_{\text{Li}}}{\rho_{\text{Li}}}\text{+}\frac{\text{1}-{\text{w}}_{\text{Li}}}{\rho_{\text{LiB}}})-\mu_{{\text{H}}_{2}\text{O}}}{\mu_{{\text{H}}_{2}\text{O}}}\\ \;\times 1,000 \end{array}$$

In the present study, Mg and C were added to the alloy. Therefore, the derivation of Equaion (7) can be deduced based on the following consideration. It can be assumed that, at first, C, B, and Li react to form a LiBC compound, and then the remaining B and Li form LiB, and finally, the residual Li and Mg form an SS.

**Figure 5:**
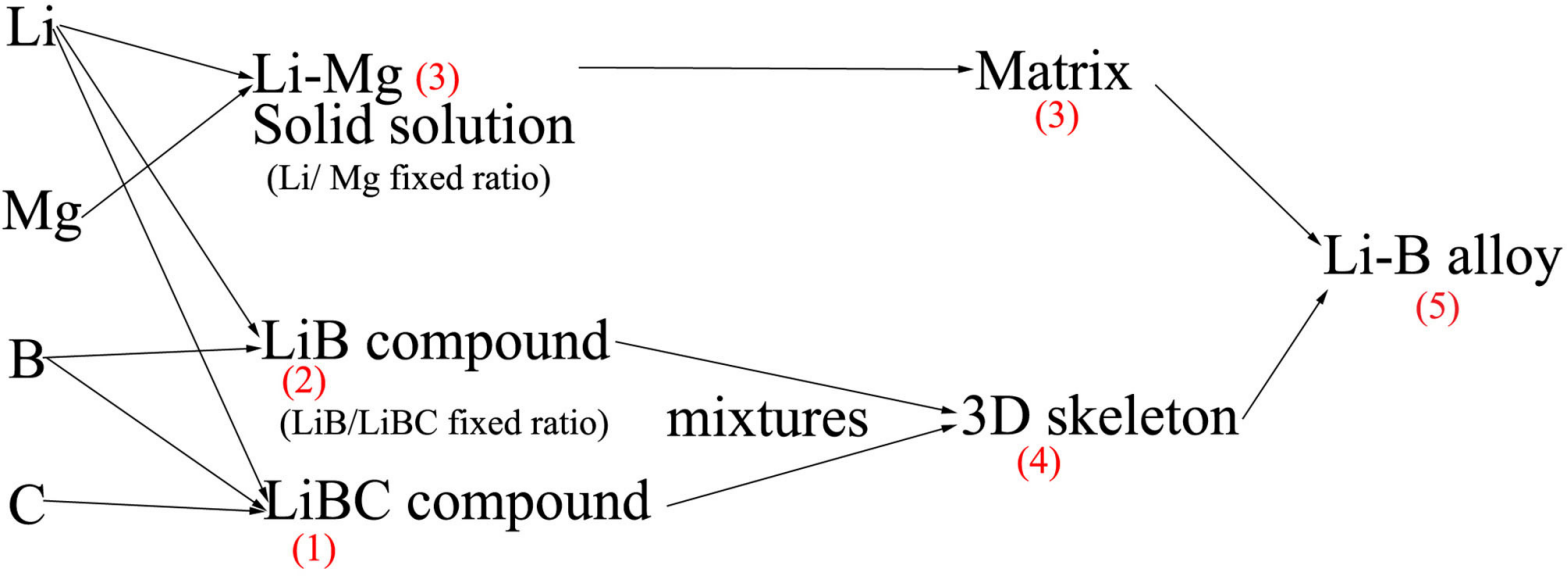
Overview of stepwise formation process of Li-B alloy.

An overview of the calculation process is given in Figure 5, whereby a stepwise method is used to calculate the relevant parameters for the three phases. Based on the elemental ratios, the relevant parameters of the hypothetical composite compound (LiB + LiBC) are first calculated. Using a volume unit for CT scanning of about 0.125 mm^3^, the proposed hypothesis is considered reasonable for the calculation. The final result may be obtained using Li-Mg (SS) and hypothetical (LiB + LiBC) to replace the Li and LiB phases, respectively, in Equation (7).

The mass percentages of LiB, LiBC, free Li and Mg can be calculated based on the data in Table 1 and section Materials and Methods. Then the MACs and densities of Li-Mg SS and the mixture (LiB + LiBC) can be calculated based on the mass percentages of free Li and Mg, LiB and LiBC, respectively, which are shown in Table 2 below.

**Table 1:** Basic elemental data.

	**Li**	**B**	**C**	**Mg**
Atomic mass	6.941	10.81	12.01	24.3
MAC (cm^2^ g^−1^ at 73 keV	0.1382	0.1506	0.1652	0.2103
Hubbell and Seltzer, 1995)				

**Table 2:** The calculated results for Li-Mg (solid solution) and other compounds.

	**Li-Mg**	**LiB**	**LiBC**	**Mixture (LiB + LiBC)**
Density (g cm^−3^)	0.569^*^	1.5#	2.134#	1.671^*^
MAC (cm^2^ g^−1^ at 73 keV)	0.1446^*^	0.1458^*^	0.1536^*^	0.1481^*^
Ratio (%)	45.2^*^	35.9^*^	18.9^*^	

* *Calculation results, #PDF 52-1033, 85-2010*.

The results for the density and MAC of the mixture at a fixed ratio are presented in Table 2. Therefore, the change in the CT value of the Li-B alloy can be used to reflect the difference of the absorption coefficient of the Li-B alloy, that is, the change of the ratio of the Li-Mg SS and the mixture. According to the change of CT value, the density and compositional change of each part of Li-B alloy ingot can be finally deduced.

Equation 8 can be obtained by substituting the respective values in Table 2 and the MAC value for H_2_O (μ_H_2_*O*_ = 0.1898 cm^−1^) into Equation (7).

(8) $$\begin{array}{r} \left[\text{Hu}\right]=\frac{13563.8}{36.2\times w_{\text{Li}}-2.8}-1,016.2 \end{array}$$

Combining Equations (4) and (8), one finds that

(9) $$\begin{array}{r} \left[\text{Hu}\right]=790.1\times \rho_{\text{Li}-\text{B}}-1,016.2 \end{array}$$

**Figure 6:**
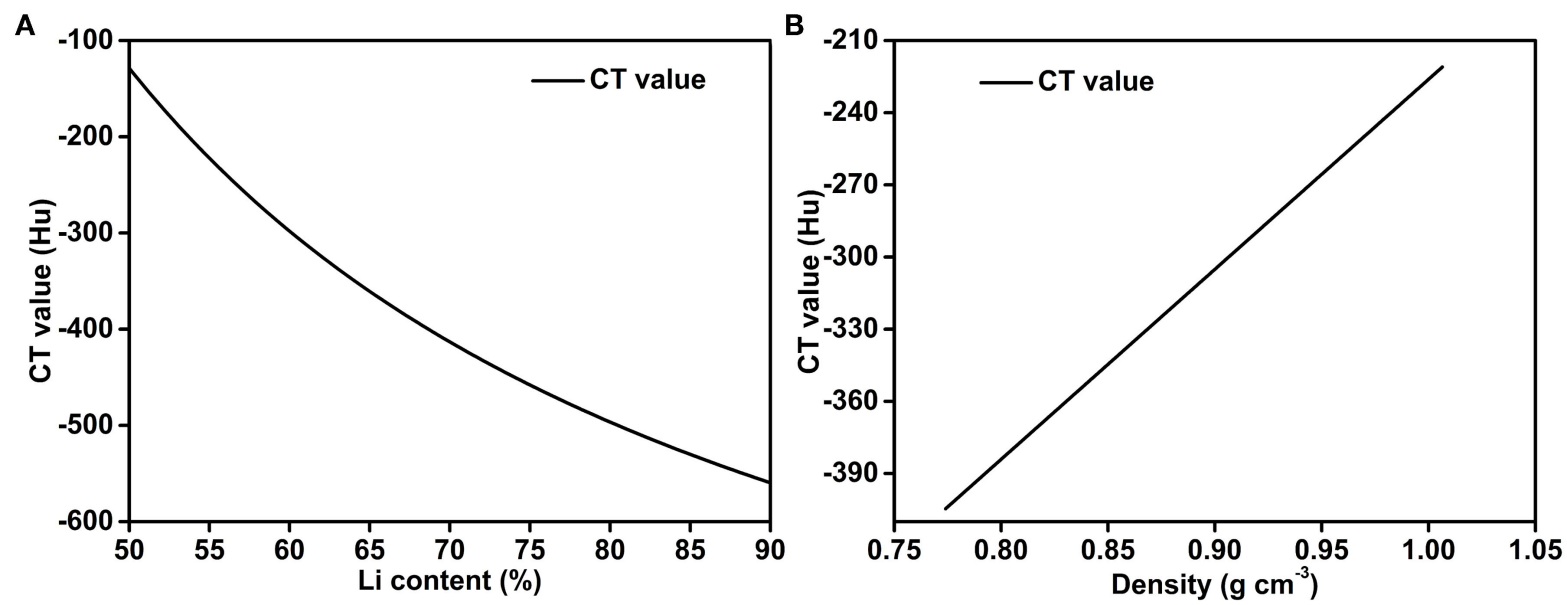
Quantitative relationships for **(A)** the CT value and Li wt.%, and **(B)** the CT value and the density of the Li-B alloy.

Hence, the Li content and the density of the Li-B alloy can be obtained from the CT values from Equation (8) and Equation (9), respectively. As shown in Figure 6, the CT value is negatively correlated with the Li content and increases in proportional to the density of the Li-B alloy.

Taking a Li content of 61% as the starting point, it can be seen from Figure 6 that when the Li content deviates by 1%, the CT values differ by 8 Hu and the density differs by 0.01 g cm^−3^, which is higher than the absolute resolution of the CT measurement (± 5 Hu) and relative resolution of the CT measurement (± 1 Hu). Therefore, the CT scanner has an error of < 0.5% with respect to measurement of the Li content. This performance is clearly superior to the performance of ICP-AES and the density test results (see later).

#### The Spatial Distribution of Li Content and Density From the CT Values

**Figure 7:**
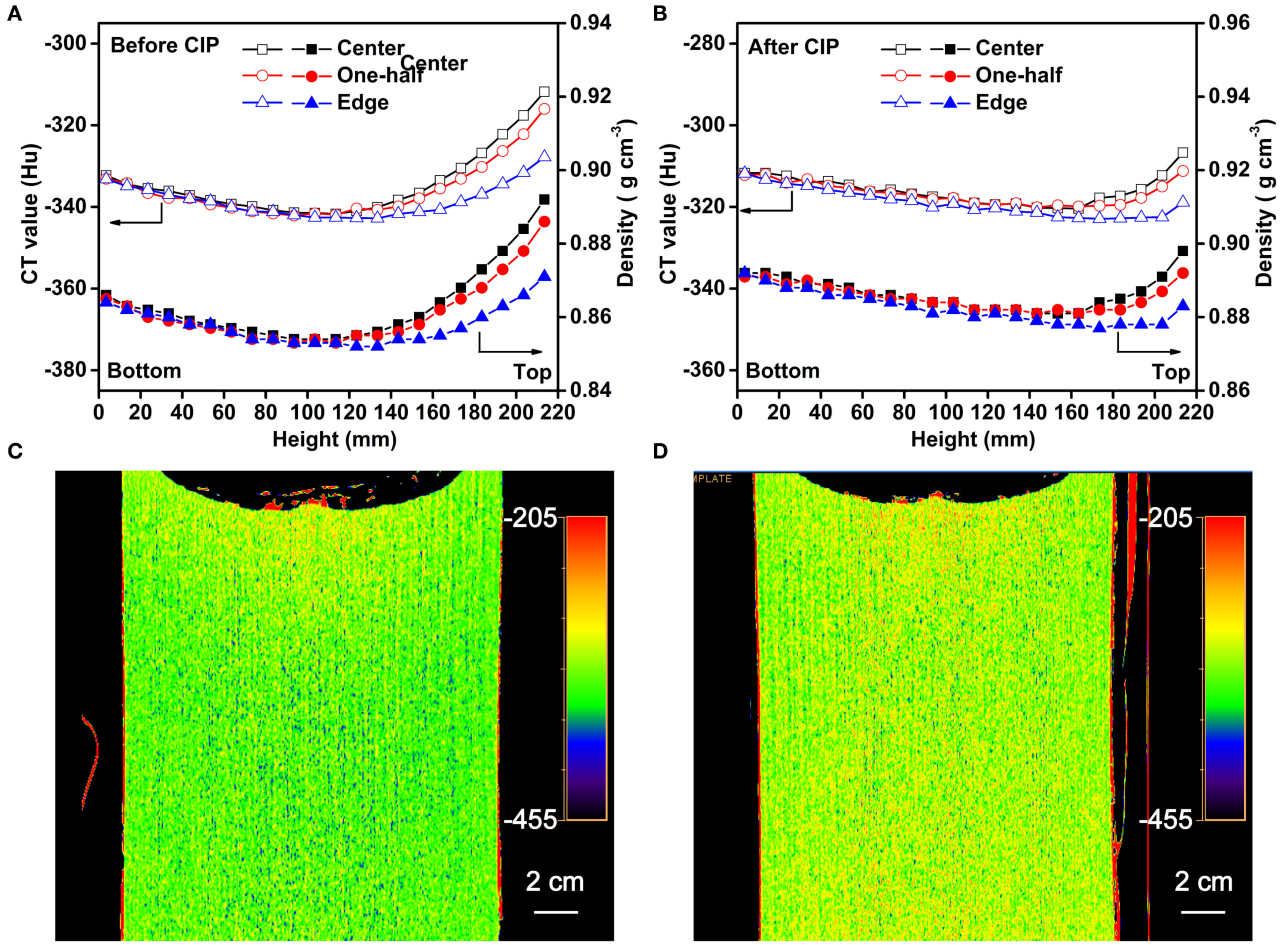
The CT values and corresponding densities of the Li-B alloy ingot **(A)** before CIP and **(B)** after CIP. The CT image **(C)** before CIP and **(D)** after CIP.

The CT data for scanning at three locations (center, 1/2 to the center, and edge positions) along the axis of the sample are presented in Figure 7A. It can be seen that the CT values and the densities of the Li-B ingot gradually decrease from bottom to top and the density reaches a minimum at the middle of the ingot. It is clear that the CT value and the density at the top-center are the highest. A comparison of the CT values and corresponding densities of the Li-B ingot before and after CIP is given in Figure 7B. The density of the ingot was increased by 2.5% on average after CIP. In addition, the increase of the density in the center and bottom locations was larger than that in the top, indicating that CIP is an effective way to remove the pores within the Li-B ingot and thus enhance density given that the existing pores are known to reduce the absorption coefficient and lower the CT value (Liu et al., 2001). According to the change of the CT value before and after CIP, the porosity (θ) of the Li-B ingot can be calculated by the formula θ = 1 − ([Hu] + 1, 000)/([Hu]′ + 1, 000), and the average porosity of the Li-B ingot is about 2.5%. Compared to the CT image before CIP (Figure 7C), the CT image after CIP (Figure 7D) exhibited a reddish tinge, which is consistent with the disappearance of pores and an increase in density.

**Figure 8:**
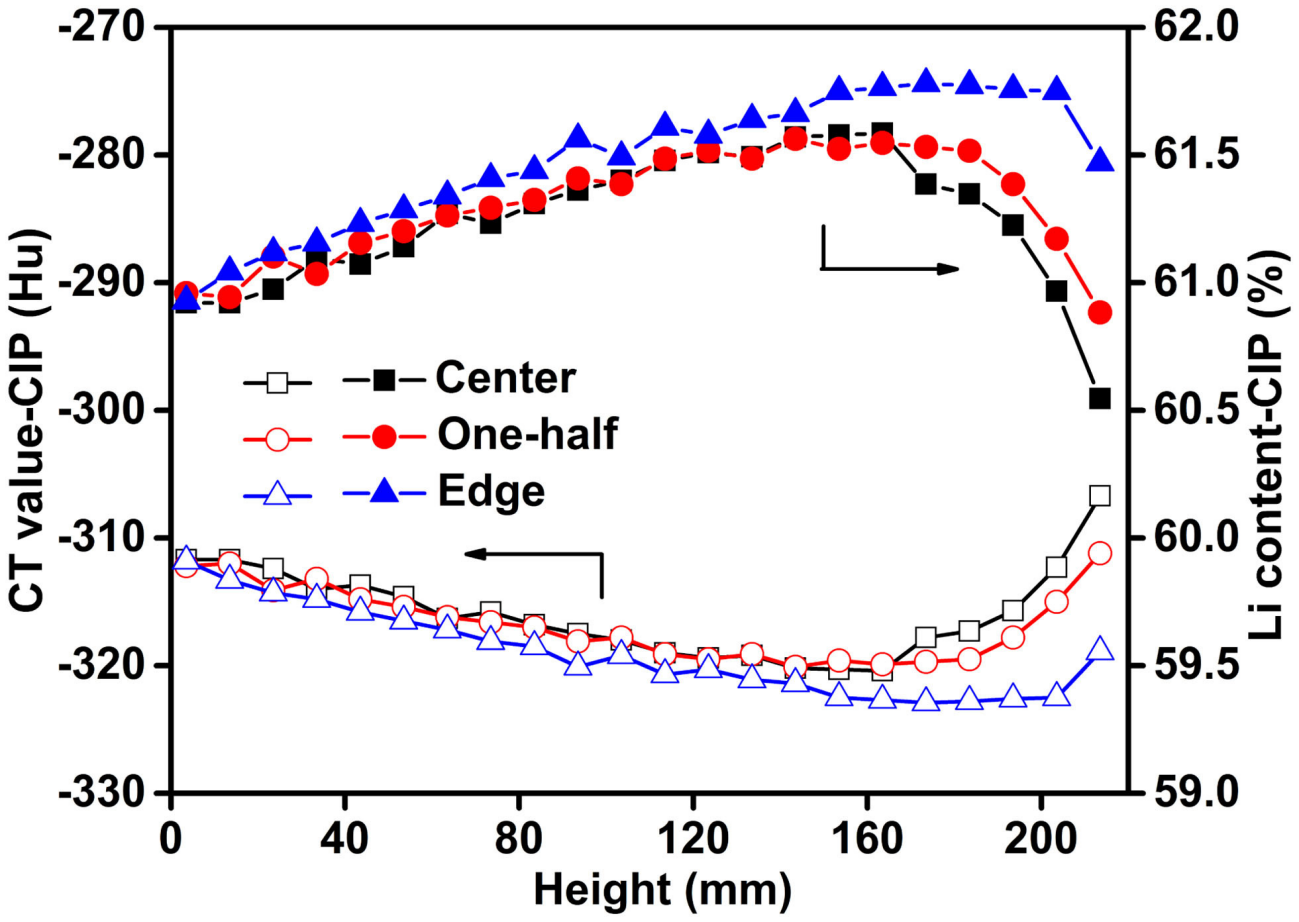
Li spatial distribution derived from the CT values.

According to Equation (8), the mass fraction of the total Li (*w*_Li_) in the Li-B alloy is negatively correlated with the CT value, which provides an effective method to determine the Li content and distribution. As shown in Figure 8, the spatial distribution of Li was not regular. The content of Li at the edge was always higher than that of the center along the whole axis. The use of CIP can decrease the number of the holes, thus the distribution characteristics for the Li content reveal the synthesis and solidification characteristics of the Li-B ingot. Given that the edge of the sample would have been first cooled during the cooling process after the reaction, the liquid Li-Mg at the edge would have solidified first, and the subsequently formed shrinkage cavities would have been then supplied by the internal unsolidified Li-Mg. During the final stage of solidification, there would have been no supplementary liquid Li-Mg available for filling of the internal Li-Mg cavities, thus a lot of shrinkage cavities would have formed, which is consistent with the color difference in Figure 7C. The change of the Li content in Li-B alloy ingot resembled a bowl due to the shrinkage in the internal regions of the ingot, which is consistent with the general metal melting characteristics. For a Li-B alloy ingot with a 61% Li content, the fluctuation in Li content was about 1%. The Li content derived from the measured CT value was close to the theoretical value, hence confirming high accuracy for the CT method.

#### Comparison of the CT Method and Other Methods

One of the traditional methods for determining the elemental content of alloys including Li-B alloys is ICP-AES. Therefore, Li-B ingots were analyzed by ICP-AES and the results for the Li content of the alloy are shown in Table 3. It can be seen that the measured Li content was significantly lower than expected. The average error calculated for Li was 5.63 wt.%. The low measured value may reflect the fact that during the sample dissolution process, a large number of bubbles were generated due to the intense reaction of the Li-B alloy with water, causing volatilization of water, a pungent odor, and possible analyte loss.

**Table 3:** Li content of the Li-B alloy (wt.%).

	**This work**			**Literature**		
	**1#**	**2#**	**3#**	**1# (Niu et al., 2014a)**	**2# (Ren et al., 2019)**	**3# (Niu et al., 2014b)**
Specified value	61	61	61	64	60	58
ICP-AES measurement	58.0	57.8	57.6	60.5	55.5	55
Error	4.9%	5.2%	5.6%	5.4%	7.5%	5.2%

**Figure 9:**
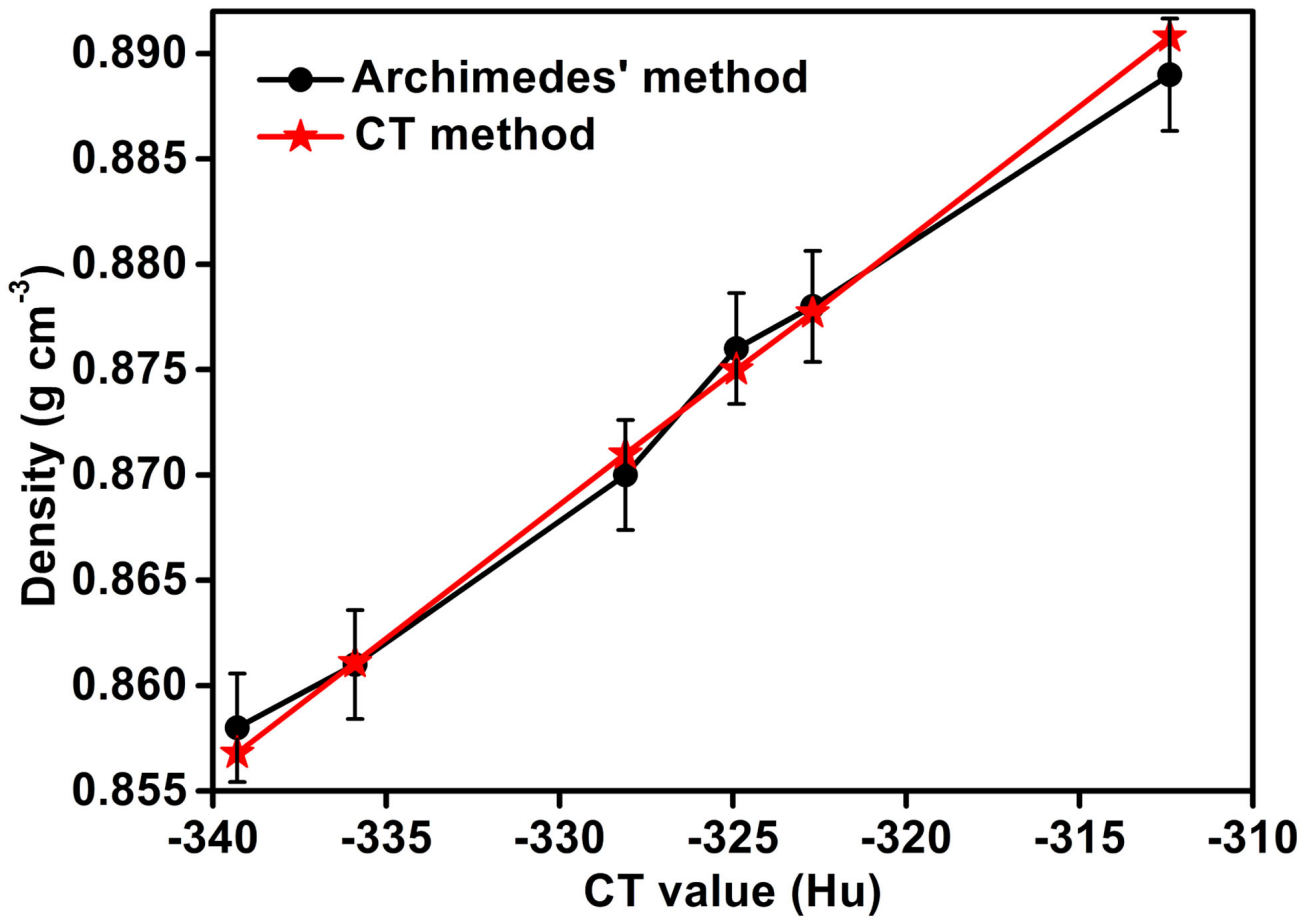
Comparison of the densities as measured by the Archimedes and the CT methods.

The densities of the Li-B ingot as measured by the CT method and Archimedes method are given in Figure 9. It can be seen that the differences in the density measured for the two methods is < 0.5%, hence confirming the effectiveness of the CT method.

This work demonstrates that CT scanning is an effective technique for spatially resolved measurement of the Li content and density of the Li-B alloy. The density and the Li content of the alloy can be readily determined according to the CT value for the specific location. Furthermore, there is no known interference effect. All the factors make the CT scanning an effective and non-destructive analysis technique for the detection of the Li content and density of the Li-B ingot.

## Conclusion

A medical CT scanner has been developed for spatially resolved measurement and imaging of Li-B alloys. The absorption of X-rays was shown to conform to the Beer-Lambert law and this permitted quantitative relationships to be established between the CT values and the Li content as well as the density of the Li-B alloy deduced. The relative accuracy of the CT method was high (± 1 HU), and the absolute accuracy for determination of the Li content was < 0.5%, which was superior to that of ICP-AES. This new method will greatly assist in advancing the fabrication process for Li-B alloys.

## Data Availability Statement

The raw data supporting the conclusions of this article will be made available by the authors, without undue reservation.

## Author Contributions

HH completed the experiments and the manuscript. CW assisted the writing of the manuscript. XZ helped conduct the experiment. ZL and LC gave guidance and suggestions. All authors contributed to the article and approved the submitted version.

## Conflict of Interest

XZ was employed by the company of Yichun Ganfeng Lithium Co., Ltd. The remaining authors declare that the research was conducted in the absence of any commercial or financial relationships that could be construed as a potential conflict of interest.
